# Engineering Furcellaran/Xanthan Gum/Beeswax Composite Films Containing Spirulina via High-Shear Homogenisation: Structural and Physicochemical Properties

**DOI:** 10.3390/ma19163391

**Published:** 2026-08-10

**Authors:** Joanna Maria Jasińska, Lesław Juszczak, Iwona Kamińska, Nikola Nowak-Nazarkiewicz, Piotr Zachariasz, Agnieszka Cholewa-Wójcik, Ewelina Jamróz

**Affiliations:** 1 Department of Chemistry, University of Agriculture in Krakow, Balicka 122, 30-149 Krakow, Poland; 2 Department of Dietetics and Food Studies, Faculty of Science & Technology, Jan Długosz University in Częstochowa, Armii Krajowej 13/15, 42-200 Czestochowa, Poland; 3 Department of Food Analysis and Evaluation of Food Quality, University of Agriculture in Krakow, Balicka 122, 30-149 Krakow, Poland; 4 Department of Botany, Physiology and Plant Protection, Faculty of Biotechnology and Horticulture, University of Agriculture in Krakow, Al. Mickiewicza 21, 31-120 Krakow, Poland; 5 Łukasiewicz Research Network-Institute of Microelectronics and Photonics, LTCC Technology, Zabłocie 39, 30-701 Krakow, Poland; 6 Department of Product Packaging, Kracow University of Economics, Rakowicka 27, 31-510 Krakow, Poland

**Keywords:** furcellaran, Spirulina, high-shear homogenisation, composite films, beeswax

## Abstract

The growing demand for sustainable and functional packaging has driven the development of biopolymer-based films prepared from food-grade components with potential suitability for edible packaging applications. This study focused on the development and characterisation of furcellaran/xanthan gum/beeswax composite films enriched with Spirulina biomass and prepared using high-shear homogenisation (HSH). The effect of Spirulina incorporation on the structural, physicochemical, mechanical and morphological properties of the films was evaluated. HSH enabled the dispersion of beeswax and Spirulina within the polysaccharide-based matrix. The FTIR spectra showed absorption bands consistent with polysaccharide-associated vibrations, including 3,6-anhydrogalactose (~930 cm^−1^) and galactose-4-sulfate (~845 cm^−1^), and indicated changes in selected functional group regions after Spirulina incorporation. XRD revealed ordered beeswax-related domains, including a reflection corresponding to a d-spacing of about 15.27 Å. Spirulina decreased the contact angle, increased WVTR approximately 2.5-fold and reduced the maximum breaking load, whereas tensile strength and elongation at break remained comparable between formulations. SEM showed a more heterogeneous morphology in Spirulina-containing films, with pores, voids and local biomass clusters. The films exhibited approximately 59% (FUR/XAN/BE) and 47% (FUR/XAN/BE/S) water solubility but formed turbid hydrogels during extraction, preventing reliable direct ABTS and CUPRAC determination. The results indicate potential for edible coatings, although further optimisation and validation are required.

## 1. Introduction

The rising global interest in sustainable agriculture and food nutrition has drawn greater attention to the development of improved packaging strategies for fresh and minimally processed food products, including fruits and vegetables. Proper packaging is essential for protecting food products from environmental threats and maintaining optimal storage conditions. In addition, growing environmental awareness and the need for eco-friendly practices to minimise waste from petrochemical-based packaging have led to extensive research efforts focused on the development of biodegradable and edible packaging materials [1]. Although packaging offers practical advantages, conventional synthetic packaging contributes substantially to environmental pollution because many petroleum-based materials are persistent and poorly biodegradable. To address these environmental issues, there is still a strong demand for new storage and packaging approaches, particularly packaging made from biodegradable and edible polymers that can be applied directly to food products [2,3]. This type of packaging offers a promising alternative for extending the shelf life of food products and reducing waste [4].

To make a packaging material suitable for edible or food-contact applications, the materials used should meet relevant food-grade requirements or be derived from natural sources approved for consumption [5]. Film-forming capability is one of the most valuable properties of polysaccharide matrices; however, their poor moisture-barrier performance, which facilitates water vapour transport, remains a major limitation. This problem can be partially reduced by using edible lipids, such as beeswax, which can form a hydrophobic phase and limit water vapour transfer [6,7,8,9]. Beeswax is an environmentally friendly coating used for food protection against dehydration. It is used as a food-grade wax additive, including under the designation E901 in the European Union, and may be considered a sustainable component of edible packaging systems. The hydrophobic character and solid lipid structure of beeswax make it useful for reducing water vapour transmission in hydrophilic biopolymer matrices [10].

The proper selection of biopolymers plays an important role in designing composites. Furcellaran, a natural polysaccharide isolated from red seaweed, is a food-related hydrocolloid used in food applications and known for its ability to form gels and films [11]. Xanthan gum, on the other hand, is a hydrocolloid frequently employed in the food industry because it modifies the viscosity and rheology of emulsions and may help prevent phase separation during film preparation [12,13]. Polysorbate 80 (Tween 80), a food-safe and edible surfactant, can be used to stabilise the aqueous and lipid phases of the film-forming emulsion. Tween 80 can decrease interfacial tension between the aqueous and lipid phases, thereby facilitating lipid dispersion and improving emulsion stability during film formation [14]. To provide additional functionality, such as colour and potential bioactivity, biopolymer matrices can be enriched with natural biomass-derived ingredients. *Arthrospira platensis*, commonly referred to as Spirulina, is an edible cyanobacterial biomass rich in proteins, phycocyanin and other antioxidant-related compounds. When incorporated into film-forming systems, Spirulina may modify the physical, chemical, optical and structural properties of the resulting films and may provide a source of bioactive compounds; however, the functionality of the final film must be verified experimentally [15,16].

High-shear homogenisation can facilitate the preparation of emulsified edible composite films by promoting the dispersion of the lipid phase, such as beeswax, within the aqueous polysaccharide matrix. In this type of system, high-shear rotor–stator homogenisation may improve emulsion stability and reduce wax droplet aggregation during solvent casting [17]. High-pressure homogenisation (HPH), including microfluidization-based approaches, has been widely applied in the development of biopolymer-based films and coatings to improve dispersion/emulsification, reduce lipid droplet or particle size, and modify the mechanical and barrier properties of the resulting materials [18,19,20]. However, HPH and high-shear homogenisation (HSH) differ substantially in their mechanisms of action. HPH relies on the passage of the dispersion through a narrow valve or interaction chamber under high pressure, where intense shear, turbulence, pressure drop and cavitation may occur, whereas HSH is primarily driven by localised shear forces and turbulence generated in a rotor–stator system [21]. In protein-containing systems, the high-energy input associated with HPH can modify the supramolecular organisation and aggregation state of proteins. For example, dynamic high-pressure homogenisation has been shown to alter the supramolecular structure of soy proteins, while repeated HPH cycles can induce both aggregation and deaggregation phenomena in myofibrillar protein dispersions [22,23]. Therefore, when processing shear- or heat-sensitive biopolymers, excessive homogenisation intensity may lead to undesired structural rearrangements, changes in surface hydrophobicity and protein aggregation, depending on the pressure level, number of passes, protein type and formulation conditions. In this context, the use of HSH rather than HPH may be relevant for developing candidate films prepared from food-grade components, containing protein-rich Spirulina biomass, because it provides intensive mixing and dispersion without exposing the system to high-pressure valve processing.

The novelty of this study lies in the formulation and characterisation of furcellaran/xanthan gum/beeswax composite films enriched with Spirulina and prepared using high-shear homogenisation. Although the individual components have been investigated previously in food-related materials, the combined effect of this polysaccharide–lipid matrix and Spirulina on the structural, physicochemical, mechanical and nanostructural properties of the resulting films remains insufficiently described. Therefore, the aim of this work is to investigate the structural and physicochemical properties of composite films based on furcellaran, xanthan gum, and beeswax, obtained via a high-shear homogenisation process with the addition of Spirulina.

## 2. Materials and Methods

### 2.1. Materials

Furcellaran type 7000 (Est-Agar AS, Kärla, Estonia) was used. It had a molecular weight of 2.951 × 10^5^, a carbohydrate content of 79.61%, a protein content of 1.18%, and a fat content of 0.24%. Beeswax (Cera Alba; 100% natural) was purchased from ECOSPA (Warsaw, Poland). Xanthan gum (100% xanthan gum, E415) was purchased from Green Essence (Pyrzyce, Poland). Spirulina powder (100% *Arthrospira platensis*) with a chlorophyll content of 12 mg/g and carotene content of 4.1 mg/g was purchased from Green Essence (Pyrzyce, Poland). Glycerol and Tween 80 were purchased from Chemland (Stargard, Poland).

#### Film Preparation

**Figure 1:**
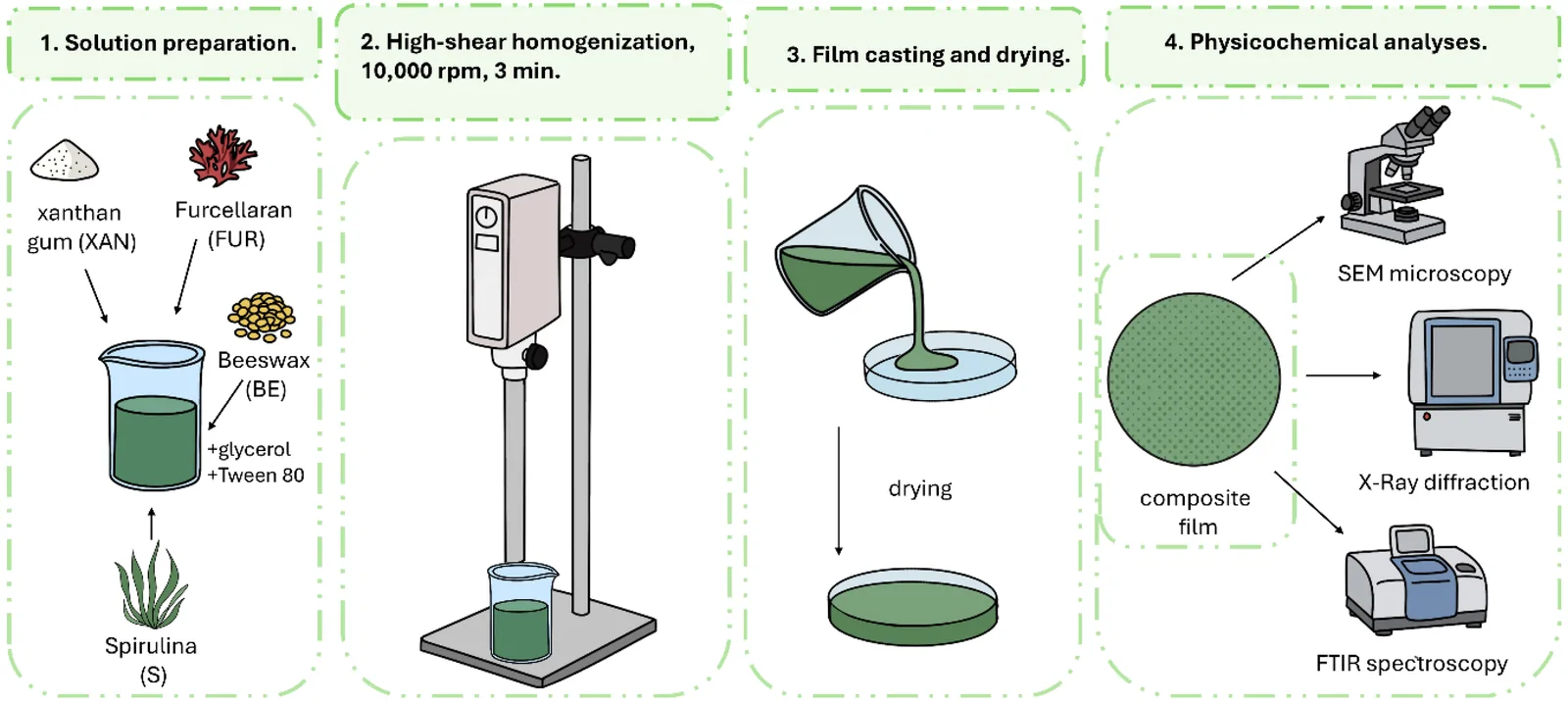
Schematic illustration of composite film preparation and characterisation.

A 1% (*w*/*v*) furcellaran (FUR) solution was prepared by dissolving the polymer in distilled water at 80 °C for 3 h under magnetic stirring. Similarly, a 2% (*w*/*v*) xanthan gum (XAN) solution was prepared at 75 °C for 3 h with continuous stirring. After complete dissolution, 100 mL of the FUR solution was combined with 25 mL of the XAN solution. Glycerol (1 mL), Tween 80 (1 mL) and beeswax (1 g) were then added to the mixture, which was heated and stirred at 90 °C for 5 min. For the Spirulina-containing films, 0.3 g of Spirulina powder was incorporated together with beeswax. The resulting film-forming dispersion was homogenised using a POLYTRON^®^ PT 2500 E high-shear rotor–stator homogeniser (Kinematica AG, Malters, Switzerland) at 10,000 rpm for 3 min to ensure the uniform dispersion of beeswax and Spirulina powder within the polysaccharide matrix. The homogenised dispersion was subsequently cast onto moulds and dried at room temperature under continuous air circulation. After complete drying, the films were carefully removed from the moulds and subjected to further physicochemical analyses. The film preparation process is presented schematically in Figure 1.

### 2.2. Film Properties

#### 2.2.1. Film Appearance, Colour and Optical Parameters

The visual appearance of the films was documented using photography. Images were captured under natural daylight with the films placed on a white paper background. The surface colour of the films was quantified using a Colour i5 spectrophotometer (X-Rite, Grand Rapids, MI, USA) under illuminant D65. Colour measurements were expressed in the CIE Lab* colour space, where L* represents lightness, a* indicates the red–green axis, and b* corresponds to the yellow–blue axis. In addition, overall colour differences (Δ*E*) were calculated to assess changes in film colouration using the following formula:

(1) $$\Delta E=\sqrt{{\left(\Delta a\right)}^{2}+{\left(\Delta b\right)}^{2}{+(\Delta L)}^{2}}$$

where Δ*E* represents the total colour difference between the reference film and the film containing Spirulina. Each sample was analysed in triplicate.

#### 2.2.2. Film Thickness

Thickness was measured to the nearest 1 μm with a Mitutoyo, No. 7327 (Kawasaki, Japan). The measurements were taken at five points, each located at least 10 mm from the edge of the film.

#### 2.2.3. Water Content and Solubility

Water content and solubility in water were evaluated according to a modified method described by Jasińska, et al. [24]. Briefly, three specimens from each film type were prepared as 2 cm × 2 cm squares and weighed with an accuracy of 0.0001 g; this mass was recorded as W1. The specimens were dried at 100 °C for 2 h, after which their dry mass was determined (W2). The dried samples were then placed in 30 mL of distilled water and stored in covered vessels for 24 h at 23 ± 2 °C. After this period, the undissolved film residues were collected and dried for a further 24 h at 23 ± 2 °C under continuous air circulation before being weighed again (W3). Water content and solubility were calculated according to Equations (2) and (3), respectively:

(2) $$\mathrm{Water}\;\mathrm{content}\;[\%]\;=\;(\frac{W1-W2}{W1})\;\times \;100$$

(3) $$\mathrm{Solubility}\;[\%]=(\frac{W2-W3}{W2})\;\times \;100$$

The measurements were run in three replications.

#### 2.2.4. Water Vapour Transmission Rate (WVTR)

The water vapour transmission rate (WVTR) of the films was determined using the gravimetric dish method according to ISO 2528:2017, with minor modifications [25,26]. Briefly, glass containers were filled with dry silica gel and sealed with the tested film samples. The exposed film area was 23.75 cm^2^, corresponding to 0.002375 m^2^. The assemblies were placed in a controlled chamber maintained at 25 °C and 75% relative humidity. The mass of each container was recorded after 24 h, and the increase in mass was used to calculate the amount of water vapour transmitted through the film. The measurements were performed in triplicate.

The WVTR values were calculated using the following equation:

(4) $$\mathrm{WVTR}\;[g/m^{2}\;\times \;d]\;=\;(\frac{\Delta m\times 24}{A\times t})$$

where WVTR denotes the water vapour transmission rate (g m^−2^ day^−1^), Δ*m* denotes the mass gain of the container during the measurement period (g), *A* denotes the exposed film area (m^2^), and *t* denotes the measurement time (h). The exposed film area was 23.75 cm^2^, corresponding to 0.002375 m^2^. In the present study, the measurement time was 24 h; therefore, Equation (4) was equivalent to WVTR = $\frac{\Delta m}{A}$.

#### 2.2.5. Water Contact Angle

The water contact angle of the films was measured using the sessile drop technique, following previously established procedures [25]. Measurements were conducted at room temperature (~23 °C) using a video-based contact angle system (OCA, Dataphysics, Filderstadt, Germany). An 8 μL droplet of deionized water was carefully placed on the film surface with a micro-injector, and the resulting droplet profile was recorded for analysis. Contact angle values were determined using five replicates of each film formulation to ensure reproducibility.

#### 2.2.6. Mechanical Properties

The mechanical properties of the films, including tensile strength (TS), elongation at break (EAB), maximum breaking load (MBL), and puncture strength (PS), were determined according to the procedures described previously [25]. According to ISO 527 standards on plastic material, tensile tests were carried out with ISO 527-1, which specifies the general principles, and ISO 527-3:1995, which specifies the test conditions for films and sheets [27,28]. Rectangular film specimens measuring 15 mm in width were stretched along their longitudinal axis until fracture or until the preset load or elongation value was reached. During the test, the values of load and elongation were recorded. Testing was carried out using a Shimadzu EZ-Test universal testing machine (Kyoto, Japan). Each film was examined three times after 24 h of conditioning at 23 °C and 50% RH. The mechanical parameters were calculated using the equations presented below:

(5) $$\mathrm{TS}\;[\mathrm{MPa}]\;=\;\frac{F_{max}}{S}$$

(6) $$\mathrm{EAB}\;[\%]=\frac{L_{f}-L_{0}}{L_{0}}\;\times \;100$$

where TS represents the tensile strength (MPa), *F_max_* is the maximum breaking load (N), S corresponds to the cross-sectional area of the film, and EAB refers to elongation at break; *L_0_* is the initial length of the specimen, and *L_f_* is the length of the specimen at fracture.

#### 2.2.7. Thermal Properties Determined by DSC

The thermal behaviour of the films was analysed using differential scanning calorimetry (DSC), following the methodology described by [29]. Measurements were conducted using a DSC 4000 calorimeter (PerkinElmer, Waltham, MA, USA). Film specimens were hermetically sealed in aluminium pans and heated from 30 to 250 °C at a rate of 10 °C/min. An empty aluminium pan was used as a reference. Thermal transition temperatures and associated enthalpy changes were determined using Proteus Analysis software v. 4.8.2 (Netzsch GmbH, Waldkraiburg, Germany), with enthalpy expressed in J/g. All measurements were performed in triplicate to ensure reproducibility.

#### 2.2.8. SEM Examination

The morphology of the samples was analysed using a Quanta 3D 200i SEM/FIB microscope (FEI, Hillsboro, OR, USA). The operating parameters included an accelerating voltage of 2 kV, a beam current of 62 pA, and a working distance of 10 mm. The samples were imaged without prior preparation. From each sample, two different spots were chosen as the most representative ones.

#### 2.2.9. X-Ray Diffraction Analysis

Structural characterisation was performed using a D8 Advance ECO diffractometer (Bruker, Karlsruhe, Germany) equipped with a Cu tube providing Cu *Kα* radiation (λ = 1.541869 Å) and operated in Bragg–Brentano geometry [30]. Wide-angle *X*-ray diffraction (WAXD) measurements were carried out at ambient temperature over 2*θ* range of 4.2–100° using the variable counting time (VCT) routine [30]. Data were processed in DIFFRAC.EVA v. 6.1 using identical settings for both patterns. The crystallinity parameter (*X*_cryst_) was evaluated across the entire 2*θ* range using an envelope background separation technique (DIFFRAC.EVA 6.1), followed by calculating an integrated diffraction area ratio:

(7) $$X_{\mathrm{cryst}}=\frac{\int_{4.2{}^{\circ}}^{100{}^{\circ}}I_{\mathrm{struct}}\left(2\theta \right)d\left(2\theta \right)}{\int_{4.2{}^{\circ}}^{100{}^{\circ}}\left(I\left(2\theta \right)-B_{0}\right)d\left(2\theta \right)}\;\times \;100\%$$

where $I_{struct}\left(2\theta \right)$ is the structural intensity obtained after separation of the diffuse-scattering envelope using a manually adjusted curvature-controlled algorithm (curvature = 31.5; threshold = 0.5), thereby defining the numerator, whereas $I\left(2\theta \right)-B_{0}$ represents the overall measured signal above the constant horizontal baseline $B_{0}$, tangent to the underside of the patterns near 2θ=100°, and defines the denominator. The resulting $X_{cryst}$ ratio is dimensionless and is reported as a percentage.

The interplanar spacings *d* were calculated from the peak positions according to Bragg’s law:

(8) nλ=2dsin θ

where n is the order of reflection, λ is the Cu *K*_α_ wavelength, and θ is angle of radiation incidence. Assuming first-order reflection (n=1), the interplanar spacing was calculated as follows:

(9) $$d=\frac{\lambda }{2sin\;\theta }.$$

Individual diffraction lines were fitted using a pseudo-Voigt function, and split Bragg reflections were deconvoluted to separate the overlapping components.

#### 2.2.10. ATR–FTIR Spectroscopy and Semi-Quantitative Spectral Analysis

FTIR spectra of the FUR/XAN/BE and FUR/XAN/BE/S films were recorded in attenuated total reflectance (ATR) mode using a Nicolet iS5 spectrometer (Thermo Fisher Scientific, Waltham, MA, USA) equipped with an iD5 ATR accessory and a zinc selenide (ZnSe) crystal. One specimen per formulation was analysed in absorbance mode over 4000–650 cm^−1^ at a resolution of 4 cm^−1^ using 16 co-added scans. A background spectrum was recorded before the sample measurements, and atmospheric correction was applied using OMNIC 9.12.1002 software (Thermo Fisher Scientific, Waltham, MA, USA).

The original OMNIC spectra were subjected to semi-quantitative analysis using a custom Python script (Python 3.13.5) with NumPy 2.3.5 [31]. The integration windows were defined prior to calculation by inspection of the experimental spectra so that each window encompassed the relevant absorption band or unresolved band envelope while limiting contributions from adjacent absorptions. Where discernible, the integration boundaries were placed at local minima surrounding the spectral feature, consistent with previously reported FTIR band-integration approaches [32,33]. Identification and assignment of the bands enclosed within these windows were guided by literature concerning natural polysaccharides, carrageenan-type galactans, beeswax, and Spirulina-derived components [34,35,36,37]. Once defined, identical spectral windows were applied to both formulations. The use of predefined spectral regions for integrated FTIR band-area analysis is consistent with earlier studies of lignocellulosic and polysaccharide-containing biological materials [32,38].

Peak positions were defined as the wavenumbers corresponding to the maximum absorbance in the original OMNIC spectrum within each window and were rounded to the nearest whole cm^−1^. Peak shifts were calculated as:

(10) $$\Delta \tilde{\nu }={\tilde{\nu }}_{B}-{\tilde{\nu }}_{A}$$

where ${\tilde{\nu }}_{A}$ and ${\tilde{\nu }}_{B}$ represent the peak wavenumbers (cm^−1^) determined for the FUR/XAN/BE and FUR/XAN/BE/S films, respectively, and $\Delta \tilde{\nu }$ denotes the corresponding change in peak position. Positive values indicate a shift towards higher wavenumbers, whereas negative values indicate a shift towards lower wavenumbers.

For integrated-area determination, a local two-point linear baseline connecting the absorbance values at the lower and upper limits of each spectral window was subtracted from the original spectrum. Negative baseline-corrected values were assigned zero contribution, and the remaining positive area was integrated using the composite trapezoidal rule after arranging the wavenumbers in ascending order. This procedure was consistent with previously reported local-baseline FTIR approaches [32,39] and trapezoidal integration of predefined spectral regions [40,41]. Identical window limits and processing parameters were used for both formulations. No spectral intensity normalisation to a reference band, maximum absorbance or total spectral area was applied. Because only one independently prepared specimen per formulation was examined and some spectral regions contained overlapping contributions from multiple functional groups, the results were treated as descriptive, semi-quantitative measures of the analysed spectral regions. They were not interpreted as absolute concentrations of individual chemical groups or subjected to statistical analysis.

#### 2.2.11. Antioxidant Analysis

An attempt was made to prepare film extracts for antioxidant analysis according to the method described by Jasińska, Michalska, Szuwarzyński, Mazur, Cholewa-Wójcik, Kopeć, Juszczak, Kamińska, Nowak and Jamróz [24] with modifications. Film samples were placed in water at 50 °C in order to obtain aqueous extracts. Methanol was also tested as an alternative solvent. However, the films did not dissolve completely. In water, the remaining film fraction formed a highly viscous, turbid hydrogel, whereas the films were insoluble in methanol. An attempt was made to separate the swollen gel phase by centrifugation at 3000× *g* for 15 min at 4 °C using refrigerated centrifuge ROTINA 380 R (Hettich, Kirchlengern, Germany). However, centrifugation did not provide effective phase separation, and no clear supernatant suitable for spectrophotometric analysis was obtained. Therefore, ABTS and CUPRAC assays were not used to quantify the antioxidant activity of the final composite films. Instead, antioxidant activity was determined for the Spirulina powder used as the functional ingredient. For this purpose, 0.3 g of Spirulina powder was mixed with 10 mL of distilled water and placed in a water bath at 50 °C. The antioxidant properties were determined using the CUPRAC and ABTS assays [42,43].

For the CUPRAC assay, the reaction mixture consisted of 1 mL of 10 mM CuCl_2_, 1 mL of 7.5 mM neocuproine in 96% ethanol, and 1 mL of 1 M ammonium acetate buffer at pH 7.0. Subsequently, 1 mL of the tested sample was added. After 30 min, the absorbance was measured at 450 nm using a Genesys 10 Vis spectrophotometer (Thermo Scientific, Waltham, MA, USA). The results were expressed as Trolox equivalents per gram of Spirulina biomass. For the ABTS assay, 3 mL of ABTS•+ solution was mixed with 1 mL of the tested sample. The absorbance was measured at 734 nm after 30 min. The results were expressed as the percentage neutralisation of ABTS•+. The analyses were performed in triplicate.

#### 2.2.12. Statistical Analysis

One-way analysis of variance (ANOVA) was performed, followed by Tukey’s HSD post hoc test to identify homogeneous groups. Statistical significance was set at *p* ≤ 0.05. The analyses were performed using Statistica 13.3 (TIBCO Software Inc., San Ramon, CA, USA).

## 3. Results and Discussion

### 3.1. Appearance and Thickness of Tested Films

The films showed good elasticity and flexibility, with a more porous, matte top surface and a smoother, shinier bottom surface (Table 1). Slight shrinkage was observed during drying, which was expected due to the relatively high polymer concentration in the film-forming dispersion. The incorporation of Spirulina into the composite film led to a significant decrease in film thickness, from 0.69 ± 0.10 mm to 0.46 ± 0.09 mm. This change may be associated with the incorporation of Spirulina powder and its interaction with the matrix during high-shear homogenisation, which could modify the internal organisation and packing of the composite network. However, this effect should not be interpreted solely as matrix densification, because the structural and barrier results indicate that Spirulina also increased heterogeneity and water vapour transmission. Therefore, the influence of Spirulina on the functional properties of polysaccharide- and beeswax-based films was evaluated further, as shown in Table 1.

### 3.2. Mechanical Properties

Biopolymer films developed as candidates for food-contact applications require adequate mechanical performance to maintain their integrity during handling, packaging, and storage, particularly when used for fruit and vegetable preservation. In the present study, high-shear homogenisation (HSH) was used as a processing step to disperse beeswax and Spirulina biomass within the FUR/XAN-based film-forming matrix.

The control films containing 0.78% BE, 0.78% FUR and 0.39% XAN exhibited a tensile strength of 2.71 ± 0.32 MPa and an elongation at break of 31.60 ± 2.86% (Table 2). These values indicate that the beeswax-containing films exhibited measurable mechanical integrity under the applied processing conditions. A similar formulation-dependent effect of beeswax was reported by Cortés-Rodríguez, et al. [44] in cassava starch/whey protein/beeswax edible coatings. The optimal formulation contained 3.17% cassava starch, 1.30% whey protein and 0.50% beeswax, and exhibited a tensile strength of 1.92 MPa, an elongation at break of 40.4% and a Young’s modulus of 42.1 MPa. These findings indicate that beeswax can be successfully incorporated into biopolymer films when its concentration is properly balanced; however, excessive lipid loading or insufficient dispersion may disrupt the continuity of the polymer matrix and negatively affect mechanical performance. A similar barrier–mechanical trade-off was observed by Petersson and Stading [45], who investigated potato starch films containing acetylated monoglyceride (Acetem) as a lipid phase. In their study, the incorporation of 10% Acetem reduced water vapour permeability, confirming the beneficial contribution of the lipid phase to moisture barrier properties. However, the same lipid addition weakened the mechanical performance of the starch films. The pure potato starch film dried at 23 °C exhibited a Young’s modulus of 2.5 ± 0.2 GPa, a tensile strength of 46.2 ± 0.8 MPa and an elongation at break of 10.3 ± 1.9%, whereas the addition of 10% Acetem reduced these values to 1.9 ± 0.3 GPa, 29.9 ± 5.5 MPa and 2.3 ± 0.5%, respectively. Thus, the incorporation of a hydrophobic lipid phase may improve water vapour barrier properties while simultaneously impairing the load-bearing capacity and deformability of the film, particularly when phase separation or discontinuities in the starch network occur. The importance of homogenisation conditions for the mechanical response of emulsified biopolymer films has also been demonstrated in chitosan-based systems. Bonilla, Atarés, Vargas and Chiralt [18] reported that the incorporation of basil or thyme essential oils into chitosan films reduced rigidity and tensile resistance while increasing extensibility, and that microfluidisation intensified these changes. For example, in chitosan–basil essential oil films containing 0.5% basil essential oil, the more intensive homogenisation treatment reduced tensile strength from 68 MPa to 32 MPa and increased elongation at break from 19% to 38%. Similarly, Flores, et al. [46] showed that the incorporation of 1.0% carvacrol into chitosan films reduced tensile strength from 39.95 ± 8.73 MPa to 23.29 ± 7.49 MPa in films homogenised using a rotor–stator system, while elongation increased from 2.45 ± 1.12% to 4.84 ± 2.20%. When the rotor–stator treatment was followed by high-pressure homogenisation, this effect was even more pronounced: tensile strength decreased from 38.46 ± 4.85 MPa to 13.05 ± 2.00 MPa, whereas elongation increased from 5.38 ± 1.74% to 15.65 ± 7.18%. These results indicate that finer and more homogeneous dispersion of the hydrophobic phase may increase film flexibility, but can also reduce cohesive strength by introducing discontinuities within the continuous chitosan matrix. The role of homogenisation in modifying the mechanical properties of chitosan films was also confirmed by Prateepchanachai, et al. [47], although in that case the effect resulted from the combined action of glycerol, NaCl and high-pressure homogenisation. The best-performing formulation, containing 25% glycerol and 15 mM NaCl and subjected to homogenisation at 10/5 MPa, reached a tensile strength of approximately 50 MPa and an elongation at break of 47%. These data show that homogenisation can either improve or weaken film mechanics depending on the formulation, the presence of plasticisers or salts, and the type and intensity of the mechanical treatment. Therefore, the literature does not support the simple interpretation that homogenisation alone improves mechanical properties; rather, it modifies the organisation of the film-forming matrix and the distribution of dispersed phases, which may lead to reinforcement, plasticisation or structural weakening.

In the present study, the addition of Spirulina produced a different but partially comparable structural effect. Unlike Acetem or essential oils, Spirulina is not a hydrophobic lipid phase and should not be interpreted as a barrier-enhancing plasticiser. Rather, it represents a particulate, protein-rich biomass that can behave as a rigid and only partially compatible filler within the polymer matrix. The incorporation of Spirulina did not substantially alter elongation at break, which remained at approximately 31–32%, and tensile strength was comparable between the films with and without Spirulina. However, the maximum breaking load decreased from 28.09 N to 19.47 N. This decrease was proportional to the reduction in film thickness and therefore primarily reflected the smaller cross-sectional area of the Spirulina-containing films. This behaviour indicates that Spirulina likely affected matrix cohesion and structural continuity rather than polymer chain mobility. The different responses of TS and MBL can be explained by the way these parameters describe film failure. TS is normalised to the cross-sectional area of the specimen, whereas MBL reflects the absolute force that the film can withstand before rupture. Consequently, the comparable TS values indicate that the load-bearing capacity per unit cross-sectional area remained similar despite the lower MBL. Nevertheless, SEM observations revealed pores, voids, local structural irregularities and fine biomass clusters in the Spirulina-containing films. Such features may interrupt the continuous polysaccharide–beeswax matrix and reduce the efficiency of stress transfer through the film during tensile loading, although their presence was not reflected in a reduction in normalised TS.

Thus, both Acetem and Spirulina can be considered as dispersed phases capable of modifying the mechanical behaviour of biopolymer films, although their mechanisms differ. Acetem, as a hydrophobic lipid phase, primarily improves moisture barrier properties but may weaken the starch network when incorporated at higher levels. Spirulina, in contrast, is a hydrophilic and particulate biomass that may introduce heterogeneous domains, interrupt polymer–polymer interactions and create local stress concentration sites. Similar effects have been reported for other Spirulina-containing biopolymer systems, including chitosan- and PLA-based films, where Spirulina incorporation reduced tensile strength and/or elongation compared with the neat polymer matrices [48,49]. Thus, although HSH likely facilitated the dispersion of beeswax and Spirulina in the film-forming system, it did not completely eliminate filler-induced discontinuities from the final film structure.

### 3.3. Water-Related Properties

The incorporation of Spirulina significantly affected the water-related properties of the composite films. The WVTR increased from 110.60 ± 35.20 g m^−2^ day^−1^ for FUR/XAN/BE to 281.96 ± 26.16 g m^−2^ day^−1^ for FUR/XAN/BE/S, representing an approximately 2.5-fold increase (Table 2). This result indicates that the addition of Spirulina reduced the resistance of the composite matrix to water vapour transport. This behaviour differs from the results reported by Ramji and Vishnuvarthanan [50], who observed improved moisture barrier properties in chitosan films containing Spirulina and MMT K10 nanoclay. In their system, the WVTR decreased from 16.24 g m^−2^ day^−1^ for the control film to 7.12 g m^−2^ day^−1^ after the addition of 0.5 g of Spirulina. This improvement was attributed to the formation of a more compact polymer network and to the presence of dispersed nanoclay platelets, which increased the tortuosity of the water vapour diffusion pathway. In contrast, the present formulation did not contain an inorganic platelet-type nanofiller, and Spirulina biomass was incorporated into a polysaccharide–beeswax matrix. Therefore, the increase in WVTR observed in this study may be attributed not only to the hydrophilic character of Spirulina but also to changes in the morphology, phase continuity and internal cohesion of the composite film. Spirulina contains proteins, polysaccharides and other polar compounds rich in hydroxyl, carbonyl, carboxyl and amino groups, which may increase the affinity of the film matrix for water molecules. This interpretation is supported by the contact angle results. The FUR/XAN/BE film showed a contact angle of 68.40 ± 1.28°, whereas the incorporation of Spirulina decreased this value to 63.11 ± 2.15° for FUR/XAN/BE/S, suggesting increased surface hydrophilicity. The decrease in contact angle can be attributed to the presence of polar functional groups originating from Spirulina proteins and polysaccharides, which may enhance the affinity of the composite matrix for water, as well as its water absorption and swelling capacity [49,51]. Similar effects have been observed in polymer composites containing microalgal biomass, where the decrease in water contact angle was attributed to the hydrophilic nature of the biomass [52]. This is also consistent with recent studies on Spirulina-containing biopolymer films, in which water-related properties such as water vapour permeability, solubility and contact angle were identified as key parameters determining the suitability of these materials for food packaging applications [49,53].

The role of waxes in modifying the surface hydrophobicity of biopolymer films was previously demonstrated by Muscat, et al. [54], who investigated high-amylose starch–glycerol films containing beeswax, candelilla wax and carnauba wax, with or without Tween 80. In their study, the neat starch film showed a contact angle of 43.7 ± 1.7°, whereas the incorporation of beeswax increased the contact angle to 44.5 ± 1.2° at 5% beeswax and to 65.6 ± 1.7° at 10% beeswax. This supports the ability of beeswax to enhance the surface hydrophobicity of polysaccharide-based films. The contact angle obtained for the FUR/XAN/BE film in the present study was comparable to that reported for the starch film containing 10% beeswax, despite the lower beeswax content in the present formulation. However, the addition of Spirulina partially counteracted this hydrophobic effect, most likely due to the introduction of hydrophilic biomass components and the increased structural heterogeneity of the composite matrix. Importantly, the increase in WVTR should not be attributed solely to surface hydrophilicity. SEM cross-sectional observations revealed pores, voids and structural irregularities in the films, with these features being more pronounced in the Spirulina-containing formulation. Such morphological discontinuities can compromise the compactness of the matrix and create preferential pathways for water vapour diffusion. In addition, Spirulina particles or local biomass clusters may disturb the continuous polysaccharide network and interfere with the homogeneous organisation of the beeswax phase. As a result, the hydrophobic barrier effect of beeswax may be partially reduced, despite its presence in the formulation.

The barrier performance of composite films depends strongly on filler dispersion, filler–matrix compatibility and the continuity of hydrophobic domains. At low or moderate concentrations, well-dispersed fillers may improve barrier properties by increasing the tortuosity of diffusion pathways. However, excessive filler loading, insufficient interfacial compatibility or particle agglomeration may produce microvoids, heterogeneous regions and local defects, thereby facilitating moisture transport through the material [55,56]. In the present study, the increased WVTR, lower contact angle and more heterogeneous SEM morphology of the FUR/XAN/BE/S films collectively suggest that Spirulina incorporation increased both the hydrophilic character and the structural heterogeneity of the matrix.

### 3.4. Thermal Properties

The addition of Spirulina did not significantly affect the thermal transition temperature; however, it affected the transition enthalpy (Δ*H*), which decreased from 48.35 to 32.07 J/g (Table 3). Similar results were observed by Gerhardt, Farias, Moura, de Almeida, da Silva, Dias, Cadaval Jr and Pinto [48] in biodegradable chitosan-based films enriched with Spirulina biomass. In that study, the peak transition temperature shifted only slightly, from 120 °C to 110 °C, whereas the enthalpy decreased markedly from 277.35 to 120.73 J/g. This decrease was attributed to modifications in intermolecular interactions and structural organisation, including disruption of polymer–polymer interactions and reduced crystallinity in the presence of Spirulina biomass filler. As a consequence, less energy was required for the thermal transition, even though the characteristic temperature of the process remained relatively unchanged. These results suggest that Spirulina affected the internal organisation of the film matrix rather than the fundamental thermal transition mechanism.

### 3.5. Antioxidant Properties

The limited extractability of the composite films prevented standard spectrophotometric antioxidant assays from being performed directly on the film extracts. The water solubility of the composite films was approximately 59% (FUR/XAN/BE) and 47% (FUR/XAN/BE/S) (Table 2), suggesting partial dissolution, possibly due to the leaching of glycerol and soluble polysaccharide fractions. However, the remaining film fraction underwent intensive swelling in water at 50 °C and formed a highly viscous, turbid hydrogel. The films were also insoluble in methanol. This gel phase could not be effectively separated by centrifugation, which prevented the preparation of a clear extract suitable for ABTS and CUPRAC measurements. This limitation is important because both ABTS and CUPRAC assays are based on spectrophotometric absorbance measurements; therefore, turbidity, suspended gel particles and non-specific absorbance from sample constituents may interfere with the optical signal and lead to unreliable antioxidant values [42,43]. This concern is particularly relevant for active packaging materials, for which antioxidant assays are often adapted from extract-based methods and may be affected by the optical properties, turbidity or incomplete separation of the tested matrix. Moreover, standardised methods for accurately quantifying the antioxidant effectiveness of packaging materials are still limited [57].

Therefore, in the present study, the antioxidant activity was determined for the Spirulina powder used as the functional ingredient rather than for the final composite films. Spirulina powder showed a high ability to neutralise the ABTS radical cation, reaching 97.46%, and exhibited antioxidant activity of 0.09 mM Trolox equivalents per gram of biomass, as determined by the CUPRAC method. This activity may be associated with the presence of bioactive Spirulina components, including phycocyanin, which is known for its antioxidant potential [58]. However, because the antioxidant activity of the final films could not be directly quantified using the applied extraction-based spectrophotometric methods, the results should be interpreted as evidence of the antioxidant potential of the incorporated Spirulina ingredient, rather than as a direct measurement of the antioxidant activity of the composite films.

### 3.6. XRD Analysis

Currently, the X-ray diffraction (XRD) method is increasingly utilised to evaluate soft, partially crystalline materials. This technique is effectively employed to distinguish macromolecular polymorphs or to ascertain characteristic distances in biopolymers or biocomposites. Both of these widely recognised measurement approaches, wide-angle X-ray scattering (WAXS) and grazing-incidence small-angle X-ray scattering (GISAXS), can be effectively applied to the investigation of soft matter, depending on the availability of scattering vector *Q*. Regarding lipids, the application of the WAXS method (where *Q* generally ranges from 0.1 to 6 Å^−1^) facilitates the analysis of short-range ordering (SRO), interplanar distances (*d*-spacing) within the crystalline fractions, or the atomic bonding region. Conversely, the GISAXS technique (*Q* < 0.1 Å^−1^) evaluates the symmetry of macromolecules, specifically the lipid bilayer of unilamellar vesicles [59].

The WAXD patterns contained a broad diffuse contribution and only a limited number of well-resolved Bragg reflections. Consequently, a comparative semi-quantitative analysis was used to evaluate structural differences between films with and without Spirulina, rather than attempting full-pattern Rietveld refinement.

**Figure 2:**
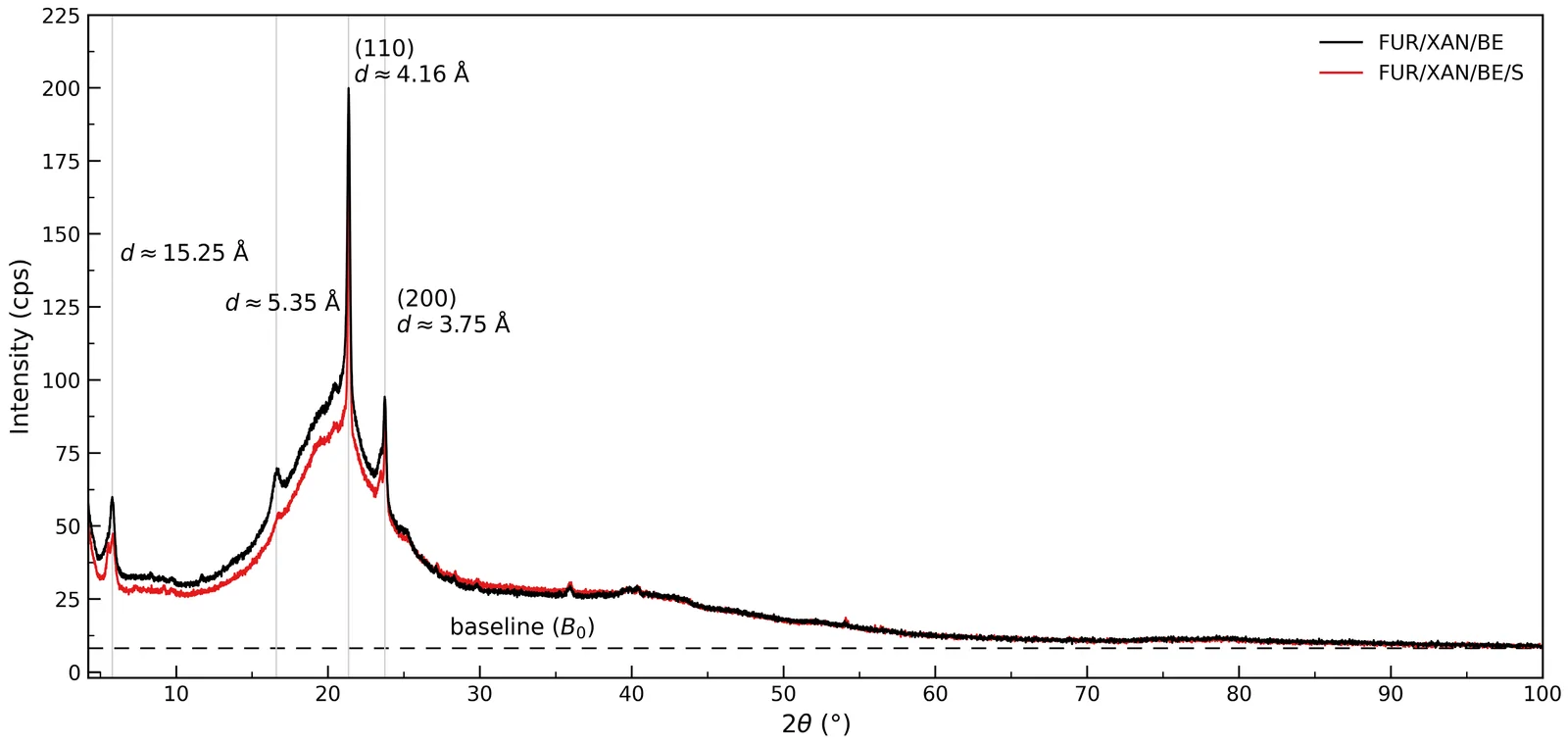
WAXD patterns of the FUR/XAN/BE and FUR/XAN/BE/S composite films.

**Figure 3:**
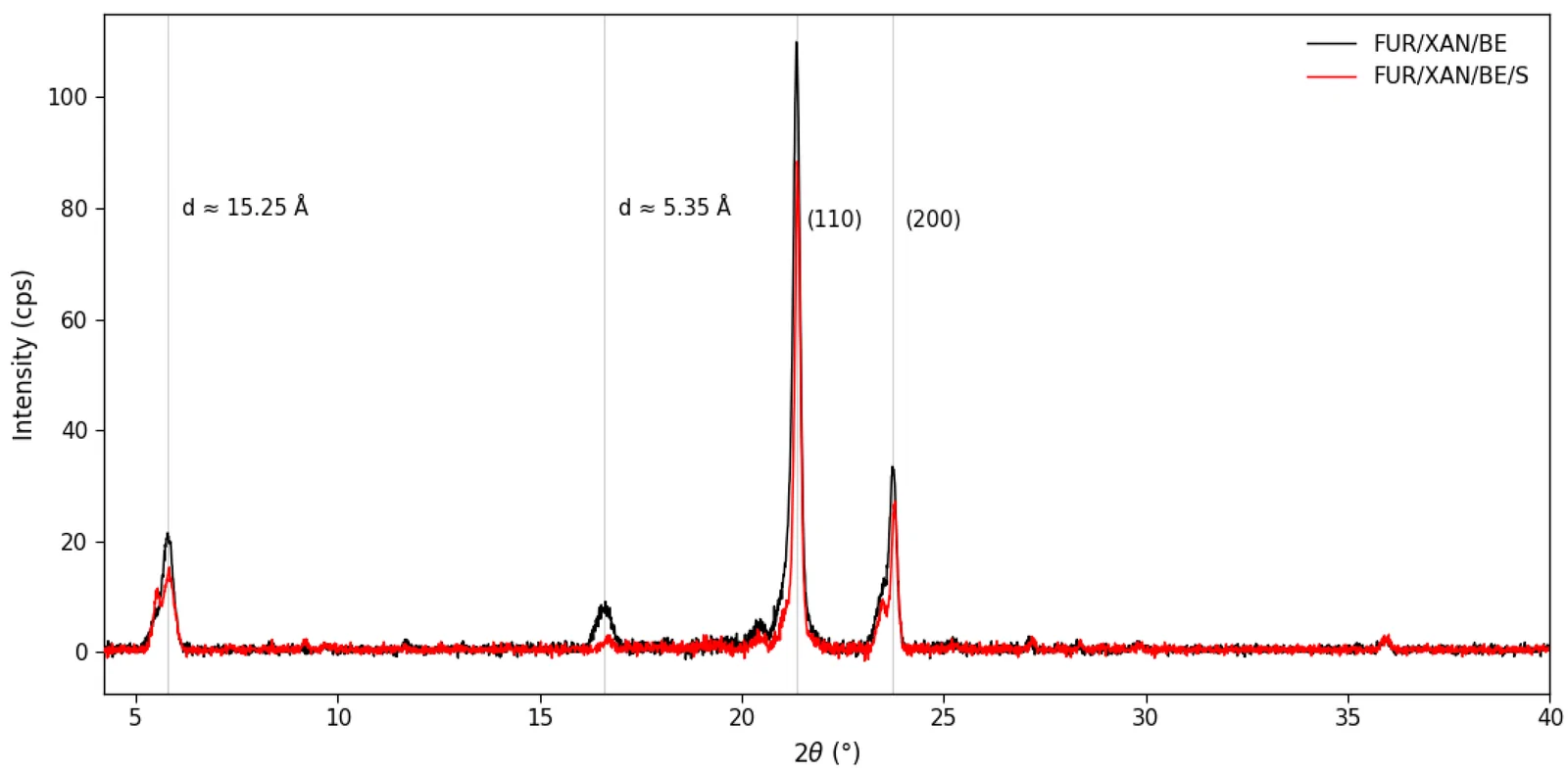
The crystalline fraction characteristic of the beeswax phase.

The full-range WAXD patterns of FUR/XAN/BE and FUR/XAN/BE/S films are shown in Figure 2, whereas Figure 3 presents the background-subtracted profiles over 2*θ* = 4.2–40° used for detailed peak analysis. Both patterns exhibited a broad diffuse contribution attributable to the organic matrix comprising furcellaran, xanthan gum, glycerol, and Tween 80. The resolved Bragg reflections arose predominantly from beeswax-associated ordered domains and included low-angle long-spacing reflections together with short-spacing lines at approximately 4.16 Å (110) and 3.75 Å (200), consistent with the local orthorhombic packing reported for long-chain aliphatic compounds [60]. The crystallinity fraction decreased from 6.1% for FUR/XAN/BE to 4.9% for FUR/XAN/BE/S. Although the ratio was obtained from the complete diffraction pattern and therefore characterises the whole film, its change is interpreted mainly in relation to the reduced contribution of beeswax-associated reflections. The pseudo-Voigt fitting results are summarised in Table 4.

Profile fitting resolved the low-angle region into two components in both formulations. For FUR/XAN/BE, the components were located at 5.428° and 5.790° (*d* = 16.28 and 15.26 Å), with integrated areas of 3.13 and 6.62 cps·°, respectively. For FUR/XAN/BE/S, the components were located at 5.509° and 5.825° (*d* = 16.04 and 15.17 Å), with integrated areas of 2.61 and 4.89 cps·°, respectively. The reflection near 5.35 Å shifted from 16.562° (*d* = 5.35 Å) to 16.653° (*d* = 5.32 Å), and its integrated area changed from 5.24 to 1.01 cps·°. The positions of the (110) reflection remained essentially unchanged (21.381° and 21.363°; *d* ≈ 4.16 Å), while the corresponding integrated areas were 20.66 and 13.10 cps·°. The (200) region was fitted with two components in each formulation. Their integrated areas were 4.31 and 7.67 cps·° for FUR/XAN/BE and 2.51 and 5.64 cps·° for FUR/XAN/BE/S.

The FWHM values of the fitted beeswax-associated components were lower after Spirulina addition. The narrower reflections may indicate reduced structural disorder and/or changes in coherent-domain size; these contributions cannot be distinguished without correction for instrumental broadening and a dedicated size-strain analysis.

Overall, Spirulina incorporation was associated with a lower crystallinity fraction, smaller integrated areas of beeswax-associated reflections, and narrower fitted components. These observations are consistent with a reorganisation of wax-associated ordered domains within the composite matrix. However, the XRD data alone do not identify a specific molecular interaction or cross-linking mechanism and do not demonstrate an improvement in macroscopic mechanical properties.

### 3.7. SEM Analysis

**Figure 4:**
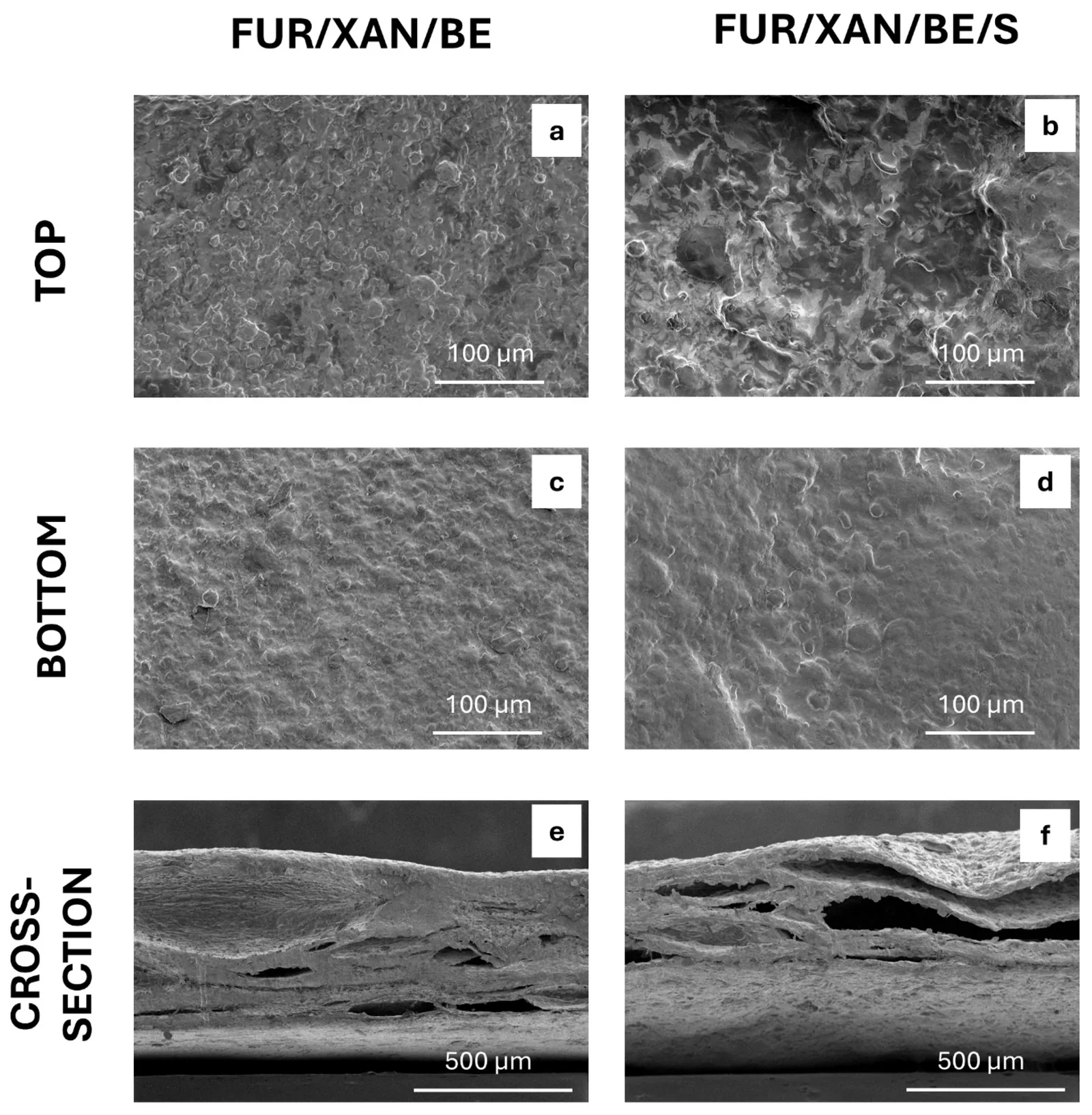
SEM micrographs of FUR/XAN/BE and FUR/XAN/BE/S films: top surface of FUR/XAN/BE (**a**), top surface of FUR/XAN/BE/S (**b**), bottom surface of FUR/XAN/BE (**c**), bottom surface of FUR/XAN/BE/S (**d**), cross-section of FUR/XAN/BE (**e**), and cross-section of FUR/XAN/BE/S (**f**). Scale bars: 100 µm for surface images and 500 µm for cross-sectional images.

Scanning electron microscopy (SEM) analysis revealed a porous and heterogeneous structure in both film formulations, with more pronounced pores, voids and structural irregularities in the Spirulina-containing films (Figure 4e,f). The presence of pores may be associated with air microbubbles retained during high-shear emulsion preparation and solvent casting [61]. However, the larger voids observed in the Spirulina-containing films may be associated with biomass inclusions, which may hinder degassing and act as additional pore nucleation sites during film formation [62]. Detailed surface examination also revealed morphological differences between the two sides of the films, which may be attributed to the solvent-casting process [63]. The upper side of the control film appeared smoother, whereas the Spirulina-containing film showed increased roughness and visible fine biomass clusters (Figure 4a,b). In contrast, the bottom surface of the films was more compact, most likely due to direct contact with the casting surface during solvent evaporation (Figure 4c,d). This asymmetry indicates that drying and casting conditions influenced the final organisation of the composite matrix. The addition of Spirulina increased surface roughness and microstructural heterogeneity, which was consistent with the incorporation of the biomass into the polysaccharide–beeswax matrix, although its distribution was not completely homogeneous at the microscale (Figure 4b). Local biomass clusters, pores and voids may disturb the continuity of the matrix and compromise the compactness of the film structure. These morphological features are consistent with the higher WVTR and lower WCA observed for the FUR/XAN/BE/S films and may contribute to local heterogeneity within the composite matrix [64,65,66,67].

### 3.8. FTIR Analysis

**Figure 5:**
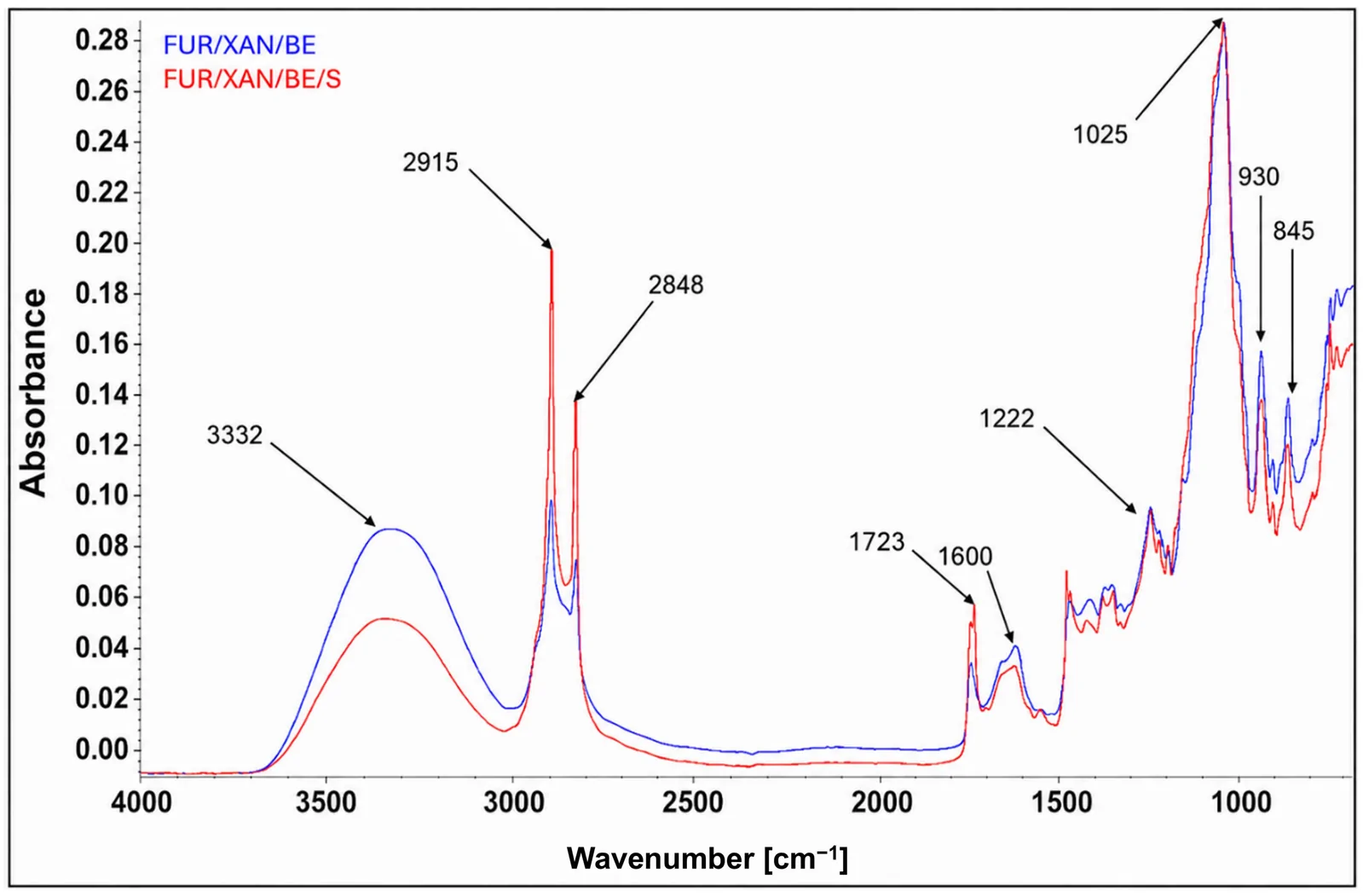
FTIR spectra of the developed composite films. Labels indicate approximate positions of characteristic absorption bands, whereas exact sample-specific peak maxima are reported in Table 5.

The FTIR spectra of the FUR/XAN/BE and FUR/XAN/BE/S composite films are presented in Figure 5. Labels in Figure 5 indicate representative positions of characteristic absorption bands, whereas the exact sample-specific peak maxima and spectral shifts are reported in Table 5. This approach was used to compare relative spectral changes after Spirulina incorporation without treating overlapping FTIR bands as absolute concentrations of individual functional groups. The bands at approximately 2916 and 2849 cm^−1^ were assigned to asymmetric and symmetric stretching vibrations of –CH_2_ groups in long aliphatic chains, which are characteristic of beeswax and other waxy lipid fractions [35,68]. As shown in Table 5, the ester C=O maximum shifted from 1735 cm^−1^ in FUR/XAN/BE to 1723 cm^−1^ in FUR/XAN/BE/S, suggesting a modified chemical environment of beeswax ester groups in the Spirulina-containing matrix. This region may also partly overlap with carbonyl vibrations associated with acetyl or pyruvyl groups of xanthan gum, which have been reported close to 1720–1730 cm^−1^ [69]. The integrated peak areas of the aliphatic C–H stretching bands increased after Spirulina incorporation. Since the beeswax concentration was kept constant in both formulations, this increase should not be interpreted as a higher absolute amount of beeswax, but rather as a change in the relative spectral contribution, distribution or surface exposure of aliphatic components within the composite matrix. In contrast, the characteristic furcellaran-related bands at approximately 925–926 and 845 cm^−1^ remained visible in both spectra, indicating retention of the polysaccharide matrix structure. These bands are commonly associated with 3,6-anhydrogalactose and galactose-4-sulfate units, respectively, and are typical of carrageenan/furcellaran-type sulfated galactans [34,70]. The band in the 1200–1265 cm^−1^ region was assigned mainly to S=O stretching vibrations of sulfate ester groups in furcellaran, although in the Spirulina-containing film this region may also overlap with amide III/C–N vibrations from proteinaceous biomass.

The 1560–1665 cm^−1^ region should be interpreted cautiously because xanthan-related carbonyl/carboxylate vibrations overlap with possible Amide I contributions from Spirulina proteins. In xanthan gum, bands around 1600 cm^−1^ are commonly attributed to carboxylate groups, whereas protein-rich microalgal biomass typically exhibits Amide I and Amide II vibrations in the regions of approximately 1600–1700 and 1500–1580 cm^−1^, respectively [69,71,72]. A weak feature around 1540 cm^−1^ became more pronounced in the Spirulina-containing film, which may support a contribution from proteinaceous biomass; however, this band was not sufficiently resolved to be used as standalone proof of protein incorporation. Overall, the FTIR data support the incorporation of Spirulina biomass and indicate changes in the local chemical microenvironment and intermolecular interaction pattern within the polysaccharide–beeswax matrix, without evidence of the formation of new covalent bonds.

**Table 5:** Semi-quantitative FTIR analysis of selected absorption bands in FUR/XAN/BE and FUR/XAN/BE/S films.

Band/Region	Integration Range (cm^−1^)	Assignment	Peak A (cm^−1^)	Peak B (cm^−1^)	Shift B-A (cm^−1^)	Integrated Peak Area FUR/XAN/BE [a.u.]	Integrated Peak Area FUR/XAN/BE/S [a.u.]	Interpretation	Refs.
O-H/N-H stretching	3000–3650	O-H stretching of polysaccharides/plasticiser; possible N-H contribution in Spirulina-containing film	3333	3345	+12	16.828	16.486	Modified hydrogen-bonding/hydration environment; no new resolved bands attributable to covalent-bond formation were observed	[37]
C-H asymmetric stretching	2885–2945	Asymmetric stretching of -CH_2_ groups in long aliphatic chains, mainly beeswax	2916	2916	0	0.581	2.458	Lipid aliphatic chains preserved; higher integrated peak area in FUR/XAN/BE/S suggests stronger relative contribution or exposure of lipid chains	[35]
C-H symmetric stretching	2815–2875	Symmetric stretching of -CH_2_ groups in long aliphatic chains, mainly beeswax	2849	2849	0	0.265	1.162	Indicates retention of beeswax-related aliphatic chains in both films.	[37,73]
C=O ester stretching	1700–1760	C=O stretching of ester groups, mainly beeswax	1735	1723	−12	0.350	1.155	Downshift after Spirulina incorporation suggests a modified chemical environment of ester carbonyls.	[74,75]
COO-/amide I region	1560–1665	Carbonyl/carboxylate vibrations of xanthan gum with possible amide I contribution from Spirulina proteins	1609	1613	+4	0.581	0.770	Overlapping region; supports possible protein contribution but should not be interpreted as a resolved amide I band alone.	[36,76]
Weak amide II region	1510–1560	Possible amide II contribution from Spirulina proteins; strongly overlapped/weak in the composite matrix	1541	1544	+3	0.005	0.060	Weak feature is more pronounced in B, but the band is not sufficiently resolved for a strong standalone conclusion.	[36]
S=O sulfate/amide III overlapping region	1200–1265	S=O stretching of sulfate esters from furcellaran; possible overlap with amide III/C-N in B	1244	1245	+1	0.289	0.348	Confirms sulfated galactan structure; changes in integrated peak area may reflect band overlap and modified local environment after Spirulina addition.	[77,78]
C-O-C/C-O polysaccharide backbone	990–1100	C-O-C/C-O stretching of glycosidic bonds in furcellaran/xanthan gum matrix	1036	1037	+1	3.490	5.652	Dominant polysaccharide band.	[79]
3,6-anhydrogalactose	905–950	Band characteristic of 3,6-anhydrogalactose units in furcellaran/carrageenan-type sulfated galactans	925	926	+1	0.664	0.986	Characteristic furcellaran-related signal retained in both films.	[34]
Galactose-4-sulfate	830–865	Band characteristic of galactose-4-sulfate units in furcellaran-type sulfated polysaccharides	845	845	0	0.257	0.405	Characteristic sulfate-substituted galactan signal retained in both films.	[80]
Long-chain CH_2_ rocking	705–735	Rocking vibrations of ordered long aliphatic chains, mainly beeswax	718	718	0	0.087	0.254	Higher integrated peak area in FUR/XAN/BE/S may indicate modified organisation or exposure of the beeswax-related phase.	[81]

Sample A corresponds to FUR/XAN/BE, whereas sample B corresponds to FUR/XAN/BE/S. Peak A and Peak B denote the wavenumber of the maximum absorbance of a given band in samples A and B, respectively. Shift B–A was calculated as ν(B) − ν(A). Integrated peak areas were calculated after local baseline correction within the selected spectral windows and are reported in arbitrary units for comparative purposes. Because several bands overlap in the composite matrix, especially in the 1700–1500 cm^−1^ region, the values should be interpreted semi-quantitatively rather than as absolute concentrations of individual functional groups.

## 4. Comprehensive Structure–Property Interpretation

Overall, the incorporation of Spirulina affected the composite films through a combination of chemical, morphological and structural mechanisms. The decrease in contact angle and the increase in WVTR suggest that Spirulina increased the hydrophilic character of the matrix, most likely due to the presence of polar protein- and polysaccharide-derived functional groups. Water-related parameters, including water vapour transport, solubility and contact angle, are among the key properties used to assess the suitability of Spirulina-containing biopolymer films for food packaging applications [53]. However, the deterioration in water vapour barrier performance cannot be attributed solely to surface hydrophilicity. SEM observations revealed a more heterogeneous morphology in the Spirulina-containing films, including larger pores, voids, local structural irregularities and fine biomass clusters. These features may compromise the compactness of the polysaccharide–beeswax matrix and create preferential pathways for water vapour diffusion. This interpretation is consistent with the general behaviour of biopolymer composite films, in which barrier and mechanical properties strongly depend on filler dispersion, filler–matrix compatibility and the continuity of the polymer phase [82]. At the same time, XRD analysis showed that the main diffraction features associated with ordered beeswax domains were still present after Spirulina incorporation, although the integrated areas of the corresponding reflections were reduced, suggesting partial disturbance of the structural organisation of the wax-containing phase. Ordered crystalline structures are commonly reported for waxes rich in long-chain aliphatic compounds [60]. This interpretation is consistent with the mechanical results: TS normalised to cross-sectional area remained comparable between the two formulations, whereas the MBL decreased after Spirulina addition. Therefore, Spirulina did not act simply as a reinforcing or plasticising component, but rather as a biomass particulate filler that modified the internal continuity of the matrix. Previous studies have shown that microalgal biomass can behave as a polymer biofiller, but its reinforcing effect depends on particle size, dispersion and interfacial adhesion with the matrix [52]. In the present films, Spirulina incorporation introduced hydrophilic domains and local microstructural discontinuities, which likely affected moisture transport and increased the structural heterogeneity of the composite matrix.

## 5. Comparison with Conventional and Biodegradable Packaging Materials

The developed FUR/XAN/BE and FUR/XAN/BE/S films were considerably thicker than most flexible packaging films reported in the literature. Their thicknesses were 0.690 ± 0.10 mm and 0.460 ± 0.09 mm, respectively, whereas commercial HDPE and LDPE films were approximately 0.100 ± 0.01 mm thick [83]. The FUR/XAN/BE/S film was also approximately 18 times thicker than OPP film, 19 times thicker than PBS film, and 13 times thicker than PBS-DLS film, for which thicknesses of 0.025 ± 0.003, 0.024 ± 0.002, and 0.035 ± 0.004 mm, respectively, were reported [84]. Similarly, PLA and PLA/melanin films were only approximately 75–80 µm thick [85]. The thickness of FUR/XAN/BE/S was closer to that of rigid thermoformed packaging, including the PET/r-PET/PET tray and the conventional APET/PE–EVOH–PE tray, with reported thicknesses of approximately 0.272 mm and 0.213 mm, respectively [86]. Although the relatively high thickness of the developed films may facilitate handling, it also indicates higher material consumption per unit area and did not result in mechanical or water vapour barrier properties comparable to those of conventional plastic packaging. The tensile strength of FUR/XAN/BE/S was 2.83 ± 0.54 MPa and did not differ significantly from that of the film without Spirulina, which reached 2.71 ± 0.32 MPa. Nevertheless, both values were substantially lower than those reported for conventional petroleum-based films. HDPE showed tensile strengths of 26–29 MPa, whereas LDPE reached 12–14 MPa [83]. Accordingly, the tensile strength of HDPE was approximately 9–10 times higher and that of LDPE approximately 4–5 times higher than the tensile strength of FUR/XAN/BE/S. An even greater difference was found in comparison with OPP, which exhibited a tensile strength of 104.36 ± 10.03 MPa nearly 37 times higher than that of FUR/XAN/BE/S [84]. The developed film was also weaker than biodegradable PBS and PBS-DLS films, with tensile strengths of 10.96 ± 0.68 and 6.36 ± 0.62 MPa, respectively, as well as PLA/melanin films, which showed tensile strengths of 40.78–45.42 MPa depending on the testing direction [84,85]. PET-based thermoformed packaging exhibited tensile strengths of approximately 49–60 MPa [86]. These comparisons show that the developed films cannot currently provide the load-bearing capacity required for applications in which the packaging is exposed to high tensile stresses during filling, transport or storage. The EAB of FUR/XAN/BE/S was 32.35 ± 1.59%, indicating moderate flexibility. This result was similar to the value of 38.16 ± 12.36% reported for PBS but was substantially lower than those of PBS-DLS and OPP, which reached 132.30 ± 25.08% and 231.84 ± 20.30%, respectively [84]. It was also markedly lower than the elongation of LDPE, reported at 420–520%, and HDPE, reported at 90–120% [83]. Conversely, the developed film was more extensible than rigid PET-based trays, for which elongation values of approximately 3.8–16% were determined, and than PHBV film, which exhibited an elongation at break of only 2.3 ± 0.6% [86,87]. Therefore, FUR/XAN/BE/S combines moderate deformability with low tensile strength. This may be sufficient for a coating or an internal packaging layer, but it is less suitable for a free-standing package subjected to substantial mechanical loads. The decrease in MBL from 28.09 ± 3.33 N for FUR/XAN/BE to 19.47 ± 3.71 N for FUR/XAN/BE/S was proportional to the decrease in film thickness. Therefore, the lower MBL primarily reflects the smaller cross-sectional area of the Spirulina-containing specimens rather than an independently demonstrated reduction in matrix cohesion. The water vapour barrier represents the principal technological limitation of the Spirulina-containing film. The incorporation of Spirulina increased WVTR from 110.60 ± 35.20 to 281.96 ± 26.16 g·m^−2^·day^−1^, corresponding to approximately a 2.55-fold increase. In comparison, OPP showed a WVTR of only 6 ± 4 g·m^−2^·day^−1^, while the value for PBS-DLS was 288 ± 26 g·m^−2^·day^−1^ and that of PBS reached 1350 ± 294 g·m^−2^·day^−1^ [84]. Thus, the WVTR of FUR/XAN/BE/S was similar to that of PBS-DLS but approximately 47 times higher than that of OPP, indicating substantially poorer moisture barrier performance. Non-metallised CPP also showed a WVTR of approximately 6.0–6.2 g·m^−2^·day^−1^, while aluminium metallisation reduced this value to approximately 0.68–0.72 g·m^−2^·day^−1^ [88]. PET-based trays evaluated by Mengozzi, Carullo, Bot, Farris and Chiavaro [86] exhibited WVTR values of approximately 3–4 g·m^−2^·day^−1^, while a SiO_x_-coated PET lid reached 0.86 ± 0.11 g·m^−2^·day^−1^ [86]. The WVTR of a PLA film containing fungal melanin was 28.20 ± 1.31 g m^−2^ day^−1^, meaning that the WVTR of FUR/XAN/BE/S was approximately ten times higher [85]. These results demonstrate that the developed material is not competitive with conventional PP- or PET-based packaging for products requiring a strong moisture barrier. However, comparisons between studies should be regarded as indicative because WVTR is strongly affected by film thickness, temperature, relative humidity, the vapour-pressure gradient, the conditioning procedure, and the measurement standard.

From an environmental perspective, the main advantage of FUR/XAN/BE/S over conventional PE, PP, and PET packaging is its predominantly renewable composition and potential suitability for edible applications. Furcellaran is obtained from algae; xanthan gum is produced by microbial fermentation; beeswax is a naturally derived lipid, and Spirulina is cyanobacterial biomass. Nevertheless, renewable origin, biodegradability, compostability, and edibility are different properties and should not be used interchangeably. Not all bio-based plastics are biodegradable; for example, bio-based PE and PP retain the same non-biodegradable polymer structure as their petroleum-derived equivalents. Conversely, some biodegradable polymers may contain fossil-derived components. PLA, PHA, PHB, PBS, starch, and several other polymers can be biodegradable, but their degradation rates depend on polymer composition and environmental conditions. PLA, for example, generally requires controlled aerobic composting conditions involving elevated temperatures and humidity, because it degrades slowly under ordinary environmental conditions [89]. The renewable composition of FUR/XAN/BE/S therefore represents a potential sustainability advantage, but its actual environmental superiority should be experimentally verified. Standardised biodegradation or compostability testing is necessary because the degradation behaviour of the complete composite may differ from that of its individual components. Similarly, the term “edible film” should be supported by food-contact safety and migration testing, as well as sensory evaluation and consumer-acceptance studies. Reviews of biopolymer food packaging emphasise that commercialisation depends not only on biodegradability but also on barrier performance, mechanical resistance, processability, scalability, safety, and cost [90].

Overall, the developed FUR/XAN/BE/S film should not currently be presented as a direct replacement for high-strength and high-barrier HDPE, LDPE, OPP, CPP, or PET packaging. Its more realistic potential lies in its use as a candidate food-contact coating, an internal functional layer, a food separator, or a packaging material for products with low moisture sensitivity and limited mechanical requirements. To demonstrate practical superiority, future work should focus on optimising the Spirulina concentration, reducing WVTR, improving the continuity of the composite structure, verifying antioxidant activity in the finished film, determining component retention, performing standardised biodegradation and migration tests, and evaluating the material in a real food-storage system. Such a balanced interpretation highlights the renewable and potentially edible characteristics of the film while clearly acknowledging that its mechanical strength, moisture barrier, and functional activity still require improvement before industrial implementation.

## 6. Conclusions and Future Perspectives

This study resulted in the development of composite films based on furcellaran, xanthan gum and beeswax and enriched with Spirulina biomass. High-shear homogenisation was used as a processing step to disperse beeswax and Spirulina within the film-forming matrix. FTIR analysis confirmed the presence of characteristic functional groups of the polysaccharide matrix, including bands associated with 3,6-anhydrogalactose and galactose-4-sulfate units, while the semi-quantitative spectral comparison indicated changes in the chemical microenvironment of selected functional groups after Spirulina incorporation. XRD analysis showed the presence of ordered beeswax-related domains, including a low-angle reflection corresponding to a d-spacing of approximately 15.27 Å and reflections assigned as (110) and (200). The incorporation of Spirulina affected the physicochemical and structural properties of the films. Although tensile strength and elongation at break remained comparable between the two formulations, the maximum breaking load decreased after Spirulina addition, suggesting reduced effective load-bearing continuity of the composite matrix. Spirulina also increased the hydrophilic character of the films, as reflected by the lower contact angle and the approximately 2.5-fold increase in WVTR. These changes were consistent with SEM observations, which revealed a more heterogeneous morphology with pores, voids and local biomass clusters in the Spirulina-containing films. Therefore, Spirulina should be considered a particulate biomass filler that modifies the internal organisation of the polysaccharide–beeswax matrix rather than a simple reinforcing or plasticising additive. Although Spirulina biomass contains potentially bioactive compounds, their functionality in the final composite films remains to be experimentally verified. The developed FUR/XAN/BE/S films may represent a promising basis for further development of candidate coatings prepared from food-grade components, containing Spirulina-derived bioactive components. However, the antioxidant activity of the final films was not directly quantified because the films formed highly turbid, viscous hydrogels during extraction, preventing reliable spectrophotometric ABTS and CUPRAC measurements. Therefore, the antioxidant functionality of the final composite film should be verified in future studies using methods adapted to insoluble or highly swelling film matrices.

Future research should focus on optimising the Spirulina concentration and its dispersion to balance potential bioactivity with moisture barrier performance. Additional studies should include direct antioxidant and antimicrobial testing of the films, assessment of bioactive compound retention after processing and drying, migration and food-contact safety studies, sensory evaluation, biodegradability assessment and application trials on real food products. Optimisation of coating application methods, such as spraying or dipping, will also be necessary to evaluate the practical suitability of these materials under realistic food storage conditions.

## Figures and Tables

**Table 1:** Appearance, colour parameters and optical properties of FUR/XAN/BE and FUR/XAN/BE/S films.

Characterisation of Films	Type of Film	
	FUR/XAN/BE	FUR/XAN/BE/S
Appearance	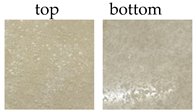	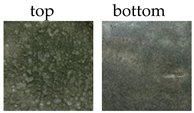
Thickness [mm]	0.69 ^a^ ± 0.10	0.46 ^b^ ± 0.09
**COLOUR PARAMETERS** (measured on the bottom side)		
L*	86.73 ^a^ ± 0.28	45.72 ^b^ ± 1.12
a*	1.71 ^a^ ± 0.08	−10.89 ^b^ ± 0.14
b*	17.22 ^a^ ± 0.37	11.39 ^b^ ± 0.20
Δ*E*	-	43.30 ± 1.13

Different lowercase superscript letters within the same row indicate significant differences according to Tukey’s HSD test (*p* ≤ 0.05).

**Table 2:** Mechanical properties and water-related characteristics of FUR/XAN/BE and FUR/XAN/BE/S films.

**MECHANICAL PROPERTIES**		
**Characterisation of Films**	**Type of Film**	
	**FUR/XAN/BE**	**FUR/XAN/BE/S**
Tensile strength (TS) [MPa]	2.71 ^a^ ± 0.32	2.83 ^a^ ± 0.54
Elongation at break (EAB) [%]	31.60 ^a^ ± 2.86	32.35 ^a^ ± 1.59
Max. breaking load (MBL) [N]	28.09 ^a^ ± 3.33	19.47 ^b^ ± 3.71
Puncture strength, (PS) [N]	4.99 ^a^ ± 0.59	3.71 ^a^ ± 0.71
**WATER PROPERTIES**		
**Characterisation of films**	**Type of film**	
	**FUR/XAN/BE**	**FUR/XAN/BE/S**
Solubility [%]	59.39 ^a^ ± 4.85	46.74 ^b^ ± 4.16
Water content [%]	9.71 ^a^ ± 0.35	6.36 ^b^ ± 0.25
Water vapour transmission rate (WVTR) (g/m^2^·d)	110.60 ^a^ ± 35.20	281.96 ^b^ ± 26.16
Water contact angle [°] (bottom side)	Bottom: 68.40 ^a^ ± 1.28 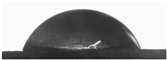	Bottom: 63.11 ^b^ ± 2.15 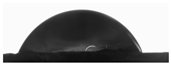

Different lowercase superscript letters within the same row indicate significant differences according to Tukey’s HSD test (*p* ≤ 0.05).

**Table 3:** Thermal properties of FUR/XAN/BE and FUR/XAN/BE/S films and antioxidant properties of Spirulina powder used as a functional ingredient in the composite films.

**THERMAL PROPERTIES**						
**Characterisation of Films**	**Type of Film**					
	**FUR/XAN/BE**			**FUR/XAN/BE/S**		
*T*_peak_ [°C]	180.25 ^a^ ± 7.91			185.11 ^a^ ± 5.55		
Δ*H* [J/g]	48.35 ^a^ ± 2.12			32.07 ^b^ ± 3.82		
**ANTIOXIDANT PROPERTIES**						
**Characterisation of the material**	**Type of material**					
	**FUR/XAN/BE**		**FUR/XAN/BE/S**		**Spirulina**	
ABTS [%]	ND		ND		97.46 ± 17.72	
CUPRAC [mM TE⋅g^−1^]	ND		ND		0.09 ± 0.0	

Different lowercase superscript letters within the same row indicate significant differences according to Tukey’s HSD test (*p* ≤ 0.05); ND, not determined.

**Table 4:** Semi-quantitative XRD analysis of the crystalline beeswax phase in FUR/XAN/BE and FUR/XAN/BE/S films.

Diffracted Line	2*θ* [°]	*d* [Å]	FWHM [°]	Area [cps·°]	2*θ* [°]	*d* [Å]	FWHM [°]	Area [cps·°]
	FUR/XAN/BE				FUR/XAN/BE/S			
~15.25 Å	5.428 5.790	16.28 15.26	0.387 0.339	3.13 6.62	5.509 5.825	16.04 15.17	0.227 0.297	2.61 4.89
~5.35 Å	16.562	5.35	0.477	5.24	16.653	5.31	0.282	1.01
(110)	21.381	4.17	0.186	20.66	21.363	4.16	0.154	13.10
(200)	23.446 23.732	3.79 3.75	0.292 0.201	4.31 7.67	23.512 23.747	3.78 3.75	0.184 0.192	2.51 5.64

Integrated peak areas were specified from XRD patterns after subtracting the diffuse background. The *d*-spacing was calculated using Bragg’s law with *λ* = 1.541869 Å.

## Data Availability

The original contributions presented in this study are included in the article. Further inquiries can be directed to the corresponding author.
